# Etymologia: *Coccidioides*

**DOI:** 10.3201/eid2106.ET2106

**Published:** 2015-06

**Keywords:** etymologia, *Coccidioides*, *C. immitis*, *C. posadasii*, fungi, coccidioidomycosis, soil, valley fever, United States, Central America, South America, Alejandro Posadas

***Coccidioides* [kok-sidʺe-oiʹdēs]**

A soil fungus found in the western United States and parts of Mexico and Central and South America, *Coccidioides* was discovered in 1892 by Alejandro Posadas, a medical student, in an Argentinian soldier with widespread disease. Biopsy specimens revealed organisms that resembled the protozoan *Coccidia* (from the Greek *kokkis*, “little berry”). In 1896, Gilchrist and Rixford named the organism *Coccidioides* (“resembling *Coccidia*”) *immitis* (Latin for “harsh,” describing the clinical course). Ophüls and Moffitt proved that *C. immitis* was a fungus rather than a protozoan in 1900. In 2002, *C. immitis* was divided into a second species, *C. posadasii*, after Alejandro Posadas.
